# Mechanical Aortic Valve Thrombosis Successfully Treated With Intravenous Thrombolytic Therapy: A Case Report

**DOI:** 10.7759/cureus.30393

**Published:** 2022-10-17

**Authors:** Shobha Mandal, Dipesh K Rohita, Sanjay Paudel, Alex Stroia, Benjamin McClintic

**Affiliations:** 1 Internal Medicine, Guthrie Robert Packer Hospital, Sayre, USA; 2 Internal Medicine, BP Koirala Institute of Health Sciences, Dharan, NPL; 3 Medicine, Guthrie Robert Packer Hospital, Sayre, USA; 4 Cardiology, Guthrie Robert Packer Hospital, Sayre, USA

**Keywords:** tpa, thrombolytic, echocardiography, aortic valve, thrombosis

## Abstract

Mechanical prosthetic valves, like all foreign bodies, are thrombogenic, requiring anticoagulation to avoid thrombosis and reduce the risk of catastrophic stroke. We reported a case of a 42-year-old female that developed mechanical valve thrombosis and was successfully treated with low infusion thrombolytic therapy using alteplase (tPA, tissue plasminogen activator).

## Introduction

Valvular heart disease affects an estimated 100 million persons worldwide leading to significant morbidity and mortality [1]. The current standard of care for the treatment of valvular heart disease in patients with low to intermediate surgical risk is surgical valve replacement [2]. Aortic valve stenosis is the most common type of valvular heart disease in the elderly. Various factors, like aging and congenital anomalies, can impair normal valve function, resulting in problems. However, aortic valve replacement has improved survival in patients with symptomatic severe aortic stenosis, which is the rationale for recommending treatment in these cases [3]. While effective at improving longevity in patients with valvular disease, valve replacement surgery is not without complications. Prosthetic valves, like all foreign bodies, are thrombogenic, requiring anticoagulation to avoid thrombosis and reduce the risk of catastrophic stroke [4]. In this case report, the patient had a mechanical valve thrombosis, which has a reported incidence of 0.1%-6% in left-sided heart valves [5]. This complication is even more concerning for patients who have subtherapeutic international normalized ratio (INR) values. One study examining patients diagnosed with prosthetic valve thrombosis (PVT) found that of the 80 patients in the study, 68.2% had subtherapeutic INR values [6]. Although this is a relatively rare complication, inadequate and untimely treatment can lead to severe consequences such as valvular dysfunction, deterioration of cardiac function, transient ischemic attack, stroke, pulmonary embolism, and death. Despite these detrimental outcomes, there is often a clinical discrepancy in the best treatment modality for patients presenting with signs of PVT. The most common treatment options for PVT are either fibrinolysis or surgical. Emergency surgery has a greater death rate in PVT patients than thrombolytic therapy, but the effectiveness of anticoagulation therapy has not been studied as thoroughly. Thus, the title of the best treatment is still up for debate [7]. The major complications of thrombolytic therapy are hemorrhage and embolism, with the incidence of complications varying based on the drugs and methods used for thrombolysis. The majority of bleeding complications are minor [8]. In the absence of significant bleeding tendencies such as pregnancy, postoperative state, or pericarditis, bleeding during fibrinolysis is unlikely [9-10]. This is a case report of a 42-year-old female that developed mechanical valve thrombosis and was successfully treated with low infusion thrombolytic therapy using alteplase (tPA, tissue plasminogen activator).

## Case presentation

A 42-year-old female with no significant medical history except for active cigarette smoking developed staphylococcal bacteremia leading to infective endocarditis after a c-section 12 years prior. She was successfully treated with a six weeks course of IV antibiotics. A few years later, she was found to have symptoms of shortness of breath on exertion and felt excessively tired with a constant state of exhaustion. She underwent cardiac evaluation with a transthoracic echocardiogram (TTE). Later, she underwent left heart catheterization with angiography, which showed normal coronary arteries but moderate to severe aortic stenosis with mild aortic regurgitation. She was evaluated by a cardiothoracic surgeon and underwent mechanical aortic valve placement with a #21 mm St. Jude mechanical valve.

After that, she was discharged home on chronic anticoagulation with warfarin as well as aspirin and metoprolol. She advised to follow up with cardiac rehabilitation as scheduled and with the warfarin clinic for INR monitoring. TTE one-year post-surgery revealed mild concentric left ventricular hypertrophy with upper normal left atrial size with a left ventricle ejection fraction of 60%-65% with adequately functioning mechanical aortic valve with transaortic gradient 22 mmHg, and estimated pulmonary artery systolic pressure of 29 mmHg. Due to being symptomatically stable, she did not follow up for her yearly echocardiogram the following year. However, she had difficulty keeping her INR within the therapeutic range, with her INR being subtherapeutic on various occasions. She presented with two months of chest pain in the left mid-axillary region, 5/10 in intensity, worse with exertion and bending forward, lasting for a few minutes then self-resolving, and radiating to the back. She also endorsed occasional awakening at night, dry cough, dizziness, and a feeling of lightheadedness on multiple occasions. She also noticed swelling of the leg and hand that had progressively gotten worse. She was inconsistent with warfarin adherence, missing a few days the previous month and also missing a warfarin clinic follow-up visit. She denied any other systemic complaints, recent travel, or exposure to sick and COVID-19-positive patients.

On admission, vitals include normal body temperature (36.7°C), a pulse rate of 110 beats/min, a blood pressure of 130/70 mmHg, and a respiratory rate of 16 breaths/min. A cardiovascular exam showed a jugular venous pulse approximately 5 cm above the right atrium, no carotid bruits, but positive radiation of an aortic murmur. Regular S1 and crisp, mechanical S2 were noted along with a 3/6 early-peaking systolic ejection murmur at the upper sternal border and throughout the precordium without any diastolic murmurs. An old, healed sternotomy scar was noted, with the remainder of the exam within normal limits. Her complete blood count (CBC), electrolytes, erythrocyte sedimentation rate (ESR), C reactive protein (CRP), and troponin-I were all normal. INR value was therapeutic at 2.8. Electrocardiogram (EKG) showed normal sinus rhythm with a heart rate in the 70s and borderline septal Q waves that were new compared to the previous EKG. Transthoracic echocardiography revealed mild concentric left ventricular hypertrophy and hyperdynamic left ventricle systolic function with no regional wall motion abnormalities. Left ventricle ejection fraction of 70%-75% and severe prosthetic aortic stenosis with effective orifice area by continuity equation (using 2.0 cm for left ventricular outflow tract, LVOT) of 0.98 cm2, mean transaortic gradient of 47 mmHg, and peak gradient of 84 mmHg was noted. The estimated pulmonary arterial systolic pressure was 42 mmHg which increased from 29 mmHg previously. She underwent urgent transesophageal echocardiography (TEE), which demonstrated severe stenosis of a mechanical aortic prosthesis, pannus versus thrombus formation, with mean and peak gradients across the aortic valve of 56 and 104 mmHg, respectively. A moderate elevation in estimated pulmonary artery systolic pressure of 53 mmHg was also noted. She was started on a heparin drip of 10,000 IV initially and INR was reversed with one dose of vitamin K 2.5 mg intramuscularly. Coronary CT angiography revealed a filling defect surrounding the mechanical aortic valve suture line, with pannus or thrombus formation likely, and was negative for coronary artery calcification, coronary artery disease, and acute aortic syndrome with limited visualization of mobility of the mechanical valve by metal artifact.

**Figure 1:**
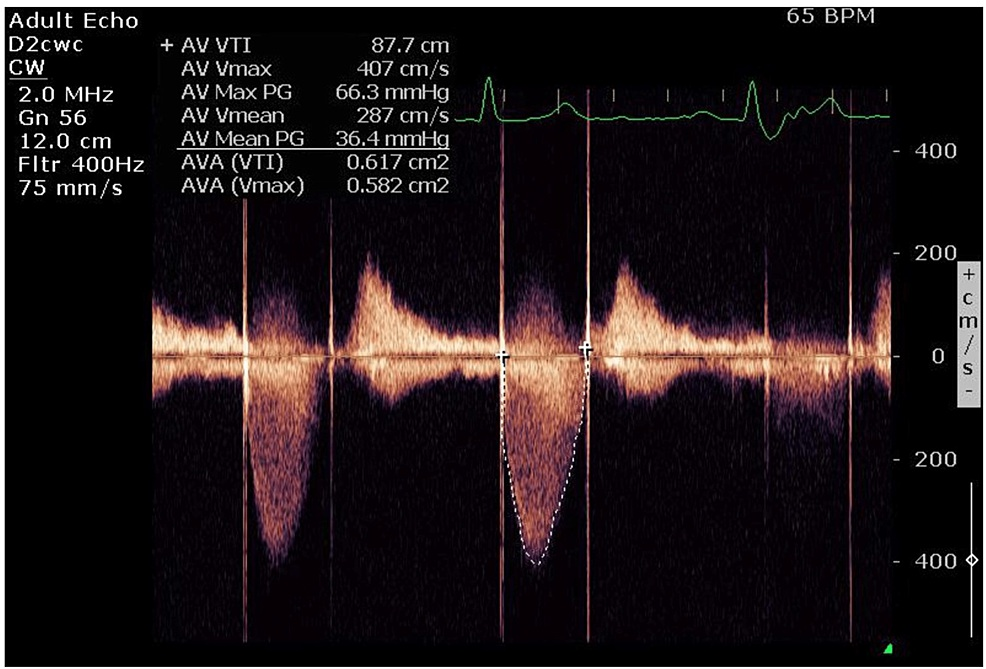
Repeat TTE post first cycle of tPA therapy showed a mean transvalvular gradient of 36 mmHg and peak transvalvular gradient of 66 mmHg. TTE, transthoracic echocardiogram; tPA, tissue plasminogen activator

The decision to proceed with medical management was made in agreement with our cardiac surgeons, and the patient was started on alteplase (tPA). A fluoroscopy was performed in a cardiac catheterization laboratory, showing an immobile anterior aortic leaflet. Thrombolytic therapy using alteplase (tPA) was given 0.04 mg/mL for six hours through a peripheral IV line, followed by an 18-h infusion of weight-based therapeutic unfractionated heparin (UFH). Re-fluoroscopy showed an opening angle between the prosthetic leaflets over 48 degrees and a closing angle of 117 degrees. This demonstrated marked improvement in the aortic valve opening compared to the previous test but was still suboptimal. Repeat TTE (Figure 1) post first cycle of tPA therapy showed a mean transvalvular gradient of 36 mmHg and a peak transvalvular gradient of 66 mmHg.

**Figure 2:**
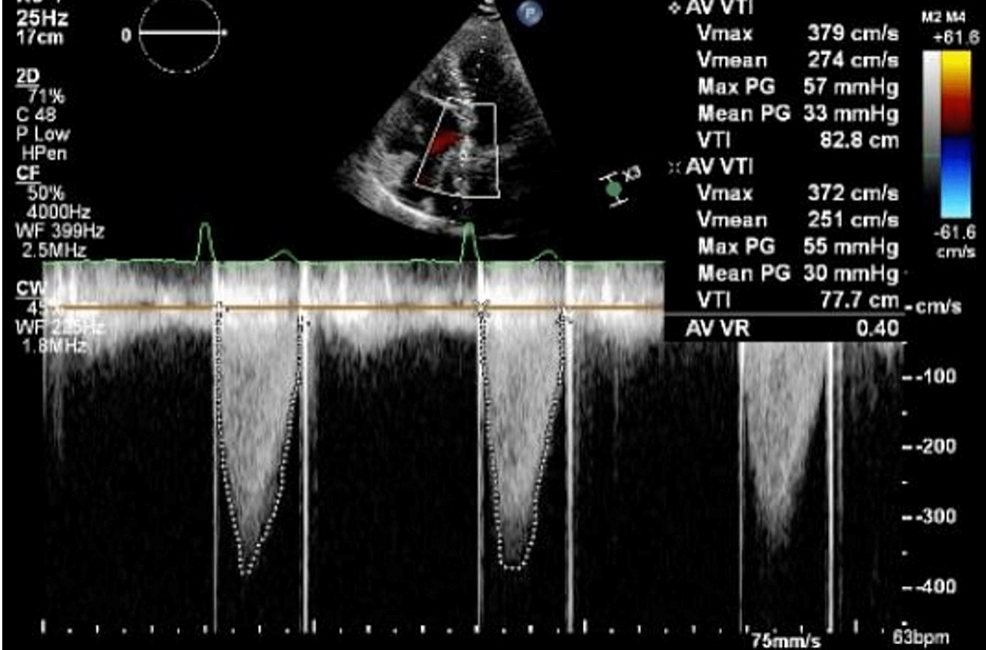
Repeat TTE post second cycle of tPA reported a mean and peak systolic gradient across the aortic valve of 39.2 and 65.1 mmHg, respectively. TTE, transthoracic echocardiogram; tPA, tissue plasminogen activator

The second dose of tPA (0.04 mg/mL over 6 h) was given, followed by an 18-h UFH infusion. Repeat TTE (Figure 2) reported a mean and peak systolic gradient across the aortic valve of 39.2 and 65.1 mmHg, respectively, improving valve movement and leaflet excursion.

The UFH overlapped with warfarin to reach the goal INR (2.5-3.5). The patient tolerated the tPA therapy without any complications, and some marginal improvements in her echocardiography gradients were noted in conjunction with a noticeable improvement in leaflet mobility via fluoroscopy. She reported significant improvement in her breathing and was bridged via Lovenox with warfarin on discharge. She was then scheduled for regular INR checkups and follow-up cardiology outpatient. A detailed discussion was undertaken regarding the importance of medication adherence with warfarin to prevent further clot formation and consultation regarding smoking cessation. On post-discharge follow-up, she reported improvement in the symptoms of shortness of breath and stated the ability to go for walks without issue. She denied any episodes of orthopnea, paroxysmal nocturnal dyspnea, palpitations, lightheadedness, or syncope.

## Discussion

Prosthetic valve thrombosis (PVT) is a rare but serious complication with high morbidity and mortality due to the potential for thromboembolism. Anticoagulation status, valve type, valve position, atrial fibrillation, and ventricular function are all factors that affect the risk of thrombosis [11]. Current studies on treatment modalities for successful management of PVT are lacking as a majority are case reports [9, 12-13] or retrospective [14]. In the absence of randomized clinical trials to guide the treatment modalities for PVT, optimal management remains controversial. The different therapeutic modalities include intensive anticoagulation therapy, thrombolytic treatment, and emergency surgery [15] with recommendations varying between organizations. Emergency valve replacement for obstructive PVT in critically ill patients without a contraindication to surgery is recommended by the European Society of Cardiology, with fibrinolysis being considered only if surgery is not an option or the risk of surgery is very high [16] . The most current 2020 suggestions from the joint American Heart Affiliation and American College of Cardiology guidelines support either slow-infusion, low-dose fibrinolytic, or crisis surgery for patients with a thrombosed left-sided mechanical prosthetic heart valve who display symptoms of valve obstacle [2]. In addition to guidelines, there have been a few retrospective studies that have investigated the effectiveness of thrombolytic therapy in the setting of PVT. One retrospective study published in 2000 evaluated 32 patients with PVT who had subtherapeutic INR [13]. The initial dose resulted in a 52% success rate, with 40% success in obstructive thrombi compared to 75% in nonobstructive thrombi [13]. After a second infusion, the success rate of both obstructive and nonobstructive thrombi was 88 [13]. Complications were seen in 15% of patients, with minor bleeding being the predominant complication. One death was reported in the study, but it was noted the patient had a 10-day-old brain embolism with no hemorrhagic infarct noted on the baseline CT [13]. Another retrospective study performed in 2020 evaluated the fibrinolytic treatment of 23 patients with valve thrombosis. The study found that 69.9% of the patients were successfully treated [7]. The mortality rate was 13% (three patients), but the study noted that two of these patients had subsequent surgeries which resulted in their death [7]. A third retrospective study evaluated 70 patients who developed mechanical valve PVT, which included 58 patients who had subtherapeutic INR [5]. For this study, 52 patients were treated with fibrinolysis and 13 patients had redo surgery based on the clinical presentations. The mortality rate for fibrinolysis was found to be 9.6% compared to redo surgery, which was 16.6% [5]. Although the limitation of these prospective studies is the small sample sizes, they indicate the potential effectiveness and safety of using fibrinolytic therapy for PVT compared to other treatment modalities, like surgical intervention. These studies also highlight the importance of more research, with greater sample sizes to better guide clinicians for appropriate treatment. In addition to prospective studies, there have been a few case reports investigating the use of thrombolytic treatment. One, in particular, was a 52-year-old female who underwent mitral valve replacement and presented with cardiac congestion symptoms seven months later. After being diagnosed with mitral PVT the patient was successfully treated with a low-dose, ultra-slow fibrinolysis. Subsequent follow-up with the patient showed her symptoms resolved, with normal mitral valve function [17]. Another case report demonstrated the successful treatment of an aortic PVT with the use of tenecteplase, a synthetic tissue plasminogen activator, in a patient who had a subtherapeutic INR. Within 48 h the patient had improvement in symptoms with improved mobility of aortic leaflets [18]. This case report exhibits the need for more research on newer fibrinolytic agents, in addition to conventional fibrinolytic.

The current literature on fibrinolytic use for PVT with patients who have subtherapeutic INR suggests it is both an effective and safe option. This case report contributes to growing evidence of using thrombolytic therapy in this patient population and the forward movement of establishing this as a standard of care to improve outcomes for patients. The findings here are crucial to advancing guidelines on the treatment of PVT with regard to the effectiveness and safety of patients undergoing fibrinolytic therapy. While there are major complications associated with thrombolytic therapy, the risk of these rare events outweighs the benefit when compared to surgical interventions. Our patient was evaluated by a cardiothoracic surgeon and was decided to treat with alteplase (tPA). She was treated with tPA for six hours followed by IV UFH for the next 18 h. Repeat fluoroscopy showed improvement in the aortic valve opening but was still suboptimal. Repeat TTE showed a mean transvalvular gradient of 36 mmHg. She was treated with the second cycle of tPA with a repeat TTE showing improvement in valve movement and leaflet excursion. She was discharged home on Lovenox bridged with warfarin. The patient reported improvement in symptoms on post-discharge follow-up with the cardiologist. Our case illustrates that TEE is helpful in the diagnosis of PVT and has the ability to continually monitor the effectiveness of treatment with thrombolysis. The rapid infusion of tPA in the management of PVT has the ability to lyse thrombus effectively [19].

## Conclusions

Treatment of prosthetic mechanical valve thrombosis can be challenging with the lack of research available comparing fibrinolysis and surgical options. Despite advances in surgical techniques and perioperative care, surgical mortality remains high, and emergent cardiac surgery is not always available. Recent evidence suggests that thrombolysis might be the therapy of choice in PVT in most settings. This case presentation demonstrated that slow infusion thrombolysis therapy with tPA can be effectively and safely used for prosthetic mechanical valve thrombosis with serial monitoring of valve thrombosis, flow gradient via transthoracic and transesophageal echocardiography.
